# Beyond Outer Nuclear Layer Predominance in Automated Hyperreflective Foci Quantification

**DOI:** 10.1167/tvst.15.5.4

**Published:** 2026-05-05

**Authors:** Henry Bair

**Affiliations:** 1 Wills Eye Hospital, Philadelphia, PA, USA. e-mail: henry.c.bair@gmail.com.

Khan and colleagues described Foci-Net, which moves hyperreflective foci (HRF) analysis from labor-intensive B-scan annotation to volumetric, layer-resolved quantification.^1^ One result, however, merits a more cautious interpretation: Because HRF volume was quantified as the proportion of total HRF volume within each retinal layer, the reported outer nuclear layer (ONL) predominance (55.37%) may partly reflect denominator effects rather than preferential biologic localization. Because these layer-specific proportions sum to 100%, an apparent increase in ONL share can arise either from true ONL enrichment or from relative depletion in neighboring layers. A layer that is thicker, expanded by drusen-related contour changes, or sampled across a larger macular footprint could accumulate more HRF volume even if its true susceptibility to harbor clinically relevant foci is no greater than that of adjacent compartments.

That distinction matters because recent work suggests that the prognostic signal of HRF is spatially specific. In intermediate age-related macular degeneration (AMD), risk of conversion to late AMD appears to depend not only on HRF burden but also on localization within the outermost retinal slabs, spanning the inner ONL to the outer neurosensory retina.^2^ Likewise, transverse mapping has shown that HRF frequency and density are not interchangeable: Lesions may be more numerous parafoveally yet more densely concentrated centrally, and eyes that progress can show a more eccentric distribution over time.^3^ Thus, where HRF are most common, where they are most concentrated, and where they are most predictive are not interchangeable questions.

Absolute HRF burden remains clinically useful, but the layerwise share of total HRF volume may not fully capture preferential localization. Foci-Net is well positioned to support analyses of where HRF are most common, where they are most concentrated, and where they are most prognostically informative. A more informative next step would be to derive geometry-adjusted metrics from the same three-dimensional segmentations: layer occupancy (HRF volume divided by available layer volume within the analyzed macular cube), an axial centroid or mean distance from the retinal pigment epithelium (RPE), and radial distance to the foveal center. Because Foci-Net already yields volumetric HRF masks, these indices may be derivable without retraining and reported alongside absolute burden. These measures would better distinguish preferential localization from simple tissue availability. They may also align more closely with disease mechanisms. Total macular HRF burden predicts the onset of persistent choroidal hypertransmission defects, but separating HRF along the RPE from intraretinal HRF improves prognostic specificity, with RPE-associated HRF carrying the strongest signal.^4^^,^^5^ A continuous RPE-referenced axial position index may therefore better reflect biologically relevant localization than coarse layer bins.

There is also a translational reason to favor normalized, retinotopic metrics. Point-to-point artificial intelligence (AI) structure–function analyses have shown that greater local HRF volume is associated with reduced mesopic retinal sensitivity, supporting the idea that precise spatial registration, rather than global burden alone, may be an important bridge between optical coherence tomography biomarkers and functional endpoints.^6^ In that light, the most important contribution of Foci-Net may be less its lesion-detection performance than its potential to transform HRF from a countable sign into a spatially calibrated phenotype.

In short, this work opens an important opportunity to move beyond asking which layer contains the most HRF volume toward asking which HRF distribution per unit tissue, per unit distance from the RPE, and per retinal locus is most prognostically informative. That shift could substantially strengthen the biological interpretability and prognostic utility of automated HRF analysis in AMD.
